# Negative and positive urgency may both be risk factors for compulsive buying

**DOI:** 10.1556/JBA.3.2014.011

**Published:** 2014-04-05

**Authors:** PAUL ROSE, DANIEL J. SEGRIST

**Affiliations:** Southern Illinois University, Edwardsville, IL, USA

**Keywords:** compulsive buying, impulsivity, positive urgency, negative urgency

## Abstract

*Background and aims:* Descriptions of compulsive buying often emphasize the roles of negative moods and trait impulsivity in the development of problematic buying habits. Trait impulsivity is sometimes treated as a unidimensional trait in compulsive buying research, but recent factor analyses suggest that impulsivity consists of multiple components that are probably best treated as independent predictors of problem behavior. In order to draw greater attention to the role of positive moods in compulsive buying, in this study we tested whether negative urgency (the tendency to act rashly while in negative moods) and positive urgency (the tendency to act rashly while in positive moods) account for similar amounts of variance in compulsive buying. *Methods:* North American adults (*N* = 514) completed an online survey containing the Richmond Compulsive Buying Scale (Ridgway, Kukar-Kinney & Monroe, 2008), established measures of positive and negative urgency (Cyders et al., 2007), *ad hoc* measures of buying-specific positive and negative urgency, measures of extraversion and neuroticism obtained from the International Personality Item Pool (http://ipip.ori.org/), and demographic questions. *Results:* In several multiple regression analyses, when demographic variables, neuroticism, and extraversion were controlled, positive urgency and negative urgency both emerged as significant predictors of compulsive buying. Whether the two urgency variables were domain-general or buying-specific, they accounted for similar amounts of variance in compulsive buying. *Conclusions:* Preventing and reducing compulsive buying may require attention not only to the purchasing decisions people make while in negative states, but also to the purchasing decisions they make while in positive states.

Compulsive buying involves repeated purchasing that is so excessive it creates personal and interpersonal problems (Kellett & Bolton, 2009). Several personality characteristics correlate with compulsive buying, and one of the best-established personality correlates is impulsivity (e.g., Billieux, Rochat, Rebetez & van der Linden, 2008; Davenport, Houston & Griffiths, 2012; DeSarbo & Edwards, 1996). Recent factor analytic work suggests that impulsivity consists of at least five facets (Cyders et al., 2007; Whiteside & Lynam, 2001), a finding that raises the possibility that some facets may predict compulsive buying more strongly than others.

Billieux et al. (2008) tested four of five impulsivity facets as independent predictors of compulsive buying. In their multiple regression analysis, only negative urgency (the tendency to act rashly while in negative states) was a significant predictor. This finding underscores the role of emotion regulation (i.e., strategies for navigating mood states; Webb, Miles & Sheeran, 2012) in compulsive buying and is part of a large body of research on the links between emotion regulation and clinical problems (see Aldeo, Nolan-Hoeksema & Schweizer, 2010; Webb, Gallo, Miles, Gollwitzer & Sheeran, 2012).

Although it is well-recognized that problem behaviors often involve negative mood states, in certain contexts, positive moods might also be problematic (see Gruber, Mauss & Tamir, 2011). However, in the Billieux et al. (2008) study, positive urgency (the tendency to act rashly in positive states, which is a recently-identified fifth facet of impulsivity [Cyders et al., 2007]) was not included as a predictor of compulsive buying. Williams and Grisham (2012) included a measure of positive urgency in their compulsive buying study, and demonstrated that both negative and positive urgency correlate with compulsive buying. They did not, however, report independent effects (such as regression coefficients). A useful next step would be to examine the independent effects of positive and negative urgency, because these two predictors positively correlate (Cyders et al., 2007), and simultaneously predict other problem behaviors such as substance use (Cyders & Smith, 2008).

In the present study, we hypothesized that both positive and negative urgency would independently account for similar amounts of variance in compulsive buying. We based this prediction on the assumption that rash actions can be positively reinforced (e.g., by feeling exhilarated) regardless of whether they are spurred by negative or positive emotions (Cyders & Smith, 2008). If rash actions made in response to either positive or negative feelings are reinforced, they may be repeated and become habitual.

The survey from which we obtained our results included established measures of positive and negative urgency (Cyders et al., 2007) as well as buying-specific measures of positive and negative urgency. Including the buying-specific measures allowed us to test our hypotheses with predictors whose effect sizes could be rigorously compared, because the items in the two buying-specific measures were identical (with only the positive and negative words differing between the two scales). Moreover, the buying-specific measures increased the clinical relevance of our study: clinicians who help people with compulsive buying problems are likely to focus more strongly on emotional dysregulation that leads to excessive buying (cf. Kellett & Bolton, 2009) than emotional dysregulation that leads to problem behaviors in general.

We also included a measure of neuroticism in this study, because neuroticism correlates with negative urgency (Kaiser, Milich, Lynam & Charnigo, 2012) and compulsive buying (Andreassen et al., 2013; Otero-López & Pol, 2013). A measure of extraversion was also included because, like positive urgency, extraversion involves positive feelings (Watson & Clark, 1997), and a correlation between extraversion and compulsive buying has emerged in some studies (e.g., Andreassen et al., 2013; Murphy, Cooper, Doran & Rose, 2012).

## METHODS

### Participants

Five hundred and fourteen adults (54% female, 46% male) registered with http://www.mturk.com responded to an online survey invitation. MTurk.com allows registered workers to peruse and select computer tasks that they can complete for modest compensation (see Buhrmester, Kwang & Gosling, 2011, for a review of MTurk.com’s value for survey research).

We restricted our recruitment to participants in the USA (88%) and Canada (11%). The mean household income was within the $25,001–$50,000 (USA) range and the mean highest level of education was between the two-year and four-year college degree response options. Because they were measured on ordinal scales, household income and education were dichotomized (through median splits; 0 = low, 1 = high) prior to the data analyses.

### Measures

*Compulsive buying.* We used the Richmond Compulsive Buying Scale because of its advantages over older measures (see Ridgway et al., 2008). Participants responded to six items (e.g., ‘Others might consider me a “shopaholic”’) using 1 (strongly disagree/never) to 7 (strongly agree/very often) scales. The total scale was internally consistent (α = .84). (Each Cronbach’s alpha we report is for the present study.)

*Neuroticism.* Participants responded to a 10-item neuroticism scale (α = .92; e.g., ‘I have frequent mood swings’) from the International Personality Item Pool (http://ipip.ori.org/) on 1 (not at all true of me) to 7 (very true of me) scales.

*Extraversion.* Using 1 (not at all true of me) to 7 (very true of me) scales, participants also responded to a 10-item extraversion scale (α = .94; e.g., ‘I feel comfortable around people’) obtained from the International Personality Item Pool.

*Negative urgency.* We used the 12 items identified by Whiteside and Lynam (2001) to assess negative urgency. Participants responded to items such as ‘I often make matters worse because I act without thinking when I am upset’ using 1 (strongly disagree) to 7 (strongly agree) scales. Four items from this scale make no reference to acting on feelings: ‘I have trouble controlling my impulses,’ ‘I have trouble resisting my cravings (for food, cigarettes, etc.),’ ‘I often get involved in things I later wish I could get out of’ and ‘Sometimes I do impulsive things that I later regret’. Given our intention of comparing the independent effects of negative and positive urgency, we chose to report analyses below for two different negative urgency variables. The *original* variable was computed as it is normally, with all 12 negative urgency items (α = .94). The *pure* variable (an eight-item scale; α = .93) was computed with the four general-impulsivity items (i.e., those just mentioned) omitted.

*Positive urgency.* Participants responded to the fourteen-item positive urgency scale (α = .96; e.g., ‘I tend to lose control when I am in a great mood’) developed by Cyders et al. (2007) using 1 (strongly disagree) to 7 (strongly agree) scales.

*Positive buying urgency and negative buying urgency.* These two buying-specific forms of urgency were each assessed with four items we wrote. To facilitate valid comparisons between the two variables, the two scales (α’s > .87) differed only in their reference to positive or negative emotions. The items were: ‘I go on buying binges when I’m in a good (bad) mood,’ ‘I get the urge to go shopping when I feel very good (bad),’ ‘Shopping helps me to enhance (escape) positive (negative) feelings’ and ‘I’m more likely to buy a lot if I’ve had a pleasant (unpleasant) day’. Participants responded to the items using 1 (strongly disagree/never) to 7 (strongly agree/always) scales.

### Treatment of outliers

Before obtaining the results reported in Tables 1 and 2, we identified outliers using a 2*k*/*n* cut-off for centered leverage values (see Cohen, Cohen, West & Aiken, 2003, p. 397). Using this rigorous criterion, 3% to 5% of cases were eliminated from the multiple regressions (in Table 2) and correlations (in Table 1).

### Ethics

The study was carried out in accordance with the principles described in the Declaration of Helsinki. The Institutional Review Board of the authors’ institution approved the study. All participants provided informed consent by advancing through survey information pages (which informed participants of their rights, such as the right to skip questions) to proceed to the survey.

## RESULTS

To examine how missing data from skipped items (and, to a lesser extent, outlier screening) may have influenced our results, we compared participants with complete data to participants with incomplete data. The complete-data group consisted of cases included in Model 1 in Table 2 (*N* = 420). The incomplete-data group consisted of cases excluded from Model 1 (*N* = 94). Complete-data participants were more likely to be female (57%), *X*^2^ (1, *N* = 512) = 6.19, *p* = .01, ø = –.11, and in the high-education group (67%), *X*^2^ (1, *N* = 512) = 4.17, *p* = .04, ø = .09. They were also higher in positive urgency, *F* (1, 484) = 32.75, *p* < .001, η^2^ = .06, and positive buying urgency, *F* (1, 494) = 6.38, *p* = .01, η^2^ = .01. Thus, participants included in the analyses were more likely to be female, had more education, and had higher positive urgency and positive buying urgency scores.

Table 1 includes descriptive statistics and correlations for each variable. Although correlations among the variables are interesting, our focus was on positive and negative urgency as *independent* predictors of compulsive buying. Accordingly, we simultaneously regressed compulsive buying on positive urgency, negative urgency (the original, 12-item version of the variable), extraversion, neuroticism and several demographic variables. The results of this regression are shown in the Model 1 column of Table 2. As hypothesized, positive urgency and negative urgency were both significant predictors. Part correlations obtained for positive and negative urgency during the regression analysis were compared using a Steiger (1980) test that yielded a non-significant result, *z* = .68, *p* = .49. Thus, positive and negative urgency accounted for approximately equal portions of variance in compulsive buying.

**Table 1.** Correlations among and descriptive statistics for key study variables

	*M (SD)*	1.	2.	3.	4.	5.	6.	7.	8.	9.	10.	11.
1. Compulsive buying	13.96 (5.89)											
2. Sex	.47 (.50)	–.09^*^										
3. Age	32.97 (11.18)	–.20^*^	–.24^*^									
4. Income	.78 (.41)	.08†	–.04	.06								
5. Education	.66 (.48)	.08†	–.15^*^	.17^*^	.13^*^							
6. Neuroticism	31.63 (13.42)	.17^*^	–.07	–.18^*^	–.16^*^	–.05						
7. Extraversion	39.23 (14.50)	.03	.01	.11^*^	.16^*^	.06	–.55^*^					
8. Neg. urgency (original)	40.00 (15.58)	.43^*^	.02	–.22^*^	–.08†	–.10^*^	.58^*^	–.28^*^				
9. Neg. urgency (pure)	26.64 (11.06)	.37^*^	.02	–.24^*^	–.08†	–.10^*^	.58^*^	–.28^*^	.98^*^			
10. Pos. urgency	28.11 (13.17)	.41^*^	.24^*^	–.32^*^	–.06	–.04	.28^*^	–.11^*^	.50^*^	.48^*^		
11. Neg. buying urgency	10.24 (5.33)	.54^*^	–.17^*^	–.12^*^	.01	.11^*^	.32^*^	–.01	.40^*^	.36^*^	.29^*^	
12. Pos. buying urgency	13.17 (5.42)	.52^*^	–.07	–.19^*^	.04	.04	.17^*^	–.02	.36^*^	.32^*^	.33^*^	.53^*^

*Notes:* N’s range from 441 to 487 due to missing data and an outlier deletion procedure described in the text. Neg. urgency (original) = negative urgency scored as usual. Neg. urgency (pure) = negative urgency scored with four general impulsivity items omitted (see the text for details). Neg. buying urgency = negative buying urgency; Pos. buying urgency = positive buying urgency.

† *p* < .10; ^*^ *p* < .05.

Next, we re-ran the regression described above using a pure negative urgency variable (computed with only the eight items that refer to acting on negative feelings). The results (presented in the Model 2 column of Table 2) were similar to those obtained with Model 1. The similarly-sized part correlations for positive and negative urgency did not significantly differ, *z* = –1.16, *p* = .25.

**Table 2.** Multiple regression analyses (Dependent variable = compulsive buying)

Predictors	Model 1 β’s	Model 2 β’s	Model 3 β’s
Sex	–.17^*^	–.19^*^	–.03
Age	–.14^*^	–.15^*^	–.13^*^
Income	.06	.03	.02
Education	.10^*^	.08†	.02
Neuroticism	–.06	–.03	.02
Extraversion	.10^*^	.13^*^	.04
Negative urgency (original)	.34^*^	–	–
Negative urgency (pure)	–	.25^*^	–
Positive urgency	.27^*^	.29^*^	–
Negative buying urgency	–	–	.35^*^
Positive buying urgency	–	–	.30^*^
*R*^2^	.30	.26	.38
*F*	21.93^*^	18.29^*^	32.95^*^
*n*	420	428	442

*Notes:* For sex, the scoring was 0 = female, 1 = male. For income and education, the scoring was 0 = below the median, 1 = at or above the median. Negative urgency (pure) represents a negative urgency total score that omits four general impulsivity items (see Methods for details).

† *p* < .10; ^*^ *p* < .05.

In both Models 1 and 2, extraversion emerged as a significant predictor even though the zero-order correlation between extraversion and compulsive buying was non-significant (i.e., there was evidence of suppression). The same regression models suggested that neuroticism was not significant when the other predictors were controlled, even though there was a significant zero-order correlation between neuroticism and compulsive buying (cf. Andreassen et al., 2013; Otero-López & Pol, 2013).

We also examined the relative importance of positive and negative urgency by regressing compulsive buying scores on buying-specific positive and negative urgency (see Model 3 in Table 2). Consistent with the results of Models 1 and 2, positive buying urgency and negative buying urgency were approximately equally strong predictors of compulsive buying. The part correlations obtained for each predictor were similar in size, *z* = .52, *p* = .60. All three regression models suggest, therefore, that positive urgency is at least as important a predictor of compulsive buying as negative urgency.

## DISCUSSION

The results of this study have important implications for consumers, clinicians and consumer advocacy groups. The roles of negative urgency (Billieux et al., 2008; Williams & Grisham, 2012) and aversive emotions (Dittmar, 2004; Kellett & Bolton, 2009; Müller et al., in press) in compulsive buying have been documented, but the possible role of positive urgency has received far less attention. Indeed, no other study that we are aware of has demonstrated, as this study did, that both positive and negative urgency independently predict compulsive buying. Both predictors were significant regardless of whether measures of general or buying-specific positive and negative urgency were used.

Cyders and Smith (2008) noted that effective interventions for consumers who are high in positive urgency seem to be lacking. Perhaps the potential negative consequences of positive moods in consumption contexts are not widely recognized. Positive affect can increase urges to action (Fredrickson & Branigan, 2005) and reduce selective attention and inhibitory control (Rowe, Hirsch & Anderson, 2007). Moreover, advertising and in-store experiences are often designed to induce positive affect (Bagozzi, Gopinath & Nyer, 1999). People with compulsive buying problems, therefore, may benefit from interventions that help identify negative *and positive* affective states as potential catalysts for unwise purchases. Additionally, clinicians working with clients who buy compulsively may find that clients who buy primarily in response to positive moods require different interventions than clients who buy primarily in response to negative moods.

The conclusions drawn from the present study are constrained by the study’s design limitations. Our convenience sample was fairly large and demographically diverse, but our analyses were based on participants with complete data who were more likely to be female, well-educated, and high in positive urgency. Additional research may reveal limits in how far this study’s findings can be generalized. In the current study, items used to measure positive urgency referred to relatively broad positive states (e.g., “being in a great mood”, “excited”, “happy”; Cyders et al., 2007). Given the physiological differences that exist between different positive emotions (see Kreibig, 2010), it seems possible that different positive states (e.g., joy, contentment) may play different roles in the behavior of compulsive buyers. The relationships between specific positive mood states, typical responses to those mood states, and compulsive buying should be the subject of future research.

Although most of the measures we used in our study have shown promise in prior research, it would be reassuring if our results were replicated with different measures. The importance of replicating this study’s results is underscored by the fact that the literature on trait predictors of compulsive buying contains several inconsistent results. For example, although extraversion was a significant predictor in two of our regression models, extraversion has not always emerged as a significant predictor of compulsive buying (cf. Andreassen et al., 2013; Mowen & Spears, 1999; OteroLópez & Pol, 2013.)

## References

[B1] Aldeo A., Nolen-Hoeksema S., Schweizer S. (2010). Emotion-regulation strategies across psychopathology: A meta-analytic review. Clinical Psychology Review.

[B2] Andreassen C. S., Griffiths M. D., Gjertsen S. R., Krossbakken E., Kvam S., Pallesen S. (2013). The relationships between behavioral addictions and the five-factor model of personality. Journal of Behavioral Addictions.

[B3] Bagozzi R. P., Gopinath M., Nyer P. U. (1999). The role of emotions in marketing. Journal of the Academy of Marketing Science.

[B4] Billieux J., Rochat L., Rebetez M. M. L., van der Linden M. (2008). Are all facets of impulsivity related to self-reported buying behavior?. Personality and Individual Differences.

[B5] Buhrmester M., Kwang T., Gosling S. D. (2011). Amazon’s Mechanical Turk: A new source of inexpensive, yet high-quality, data?. Perspectives on Psychological Science.

[B6] Cohen J., Cohen P., West S., Aiken L. (2003). Applied multiple regression/correlation analysis for the behavioral sciences.

[B7] Cyders M. A., Smith G. T. (2008). Emotion-based dispositions to rash action: Positive and negative urgency. Psychological Bulletin.

[B8] Cyders M. A., Smith G. T., Spillane N. S., Fischer S., Annus A. M., Peterson C. (2007). Integration of impulsivity and positive mood to predict risky behavior: Development and validation of a measure of positive urgency. Psychological Assessment.

[B9] Davenport K., Houston J. E., Griffiths M. D. (2012). Excessive eating and compulsive buying behaviours in women: An empirical pilot study examining reward sensitivity, anxiety, impulsivity, self-esteem and social desirability. International Journal of Mental Health and Addiction.

[B10] DeSarbo W. S., Edwards E. A. (1996). Typologies of compulsive buying behavior: A constrained clusterwise regression approach. Journal of Consumer Psychology.

[B11] Dittmar H., R. Coombs (2004). Understanding and diagnosing compulsive buying. Handbook of addictive disorders: A practical guide to diagnosis and treatment.

[B12] Fredrickson B. L., Branigan C. (2005). Positive emotions broaden the scope of attention and thought-action repertoires. Cognition & Emotion.

[B13] Gruber J., Mauss I. B., Tamir M. (2011). A dark side of happiness? How, when, and why happiness is not always good. Perspectives on Psychological Science.

[B14] Kaiser A. J., Milich R., Lynam D. R., Charnigo R. J. (2012). Negative urgency, distress tolerance, and substance abuse among college students. Addictive Behaviors.

[B15] Kellett S., Bolton J. V. (2009). Compulsive buying: A cognitive-behavioural model. Clinical Psychology and Psychotherapy.

[B16] Kreibig S. D. (2010). Autonomic nervous system activity in emotion: A review. Biological Psychology.

[B17] Mowen J. C., Spears N. (1999). Understanding compulsive buying among college students: A hierarchical approach. Journal of Consumer Psychology.

[B18] Müller A., Mitchell J. E., Crosby R. D., Cao L., Johnson J., Claes L., de Zwaan M. Mood states preceding and following compulsive buying episodes: An ecological momentary assessment study. Psychiatry Research.

[B19] Murphy M., Cooper T., Doran M., Rose P., M. Cyders (2012). Impulsivity factors which predict compulsive buying. Psychology of impulsivity..

[B20] Otero-López J. M., Pol E. V. (2013). Compulsive buying and the Five Factor Model of personality: A facet analysis. Personality and Individual Differences.

[B21] Ridgway N., Kukar-Kinney M., Monroe K. B. (2008). An expanded conceptualization and a new measure of compulsive buying. Journal of Consumer Research.

[B22] Rowe G., Hirsch J. B., Anderson A. K. (2007). Positive affect increases the breadth of attentional selection. Proceedings of the National Academy of Sciences.

[B23] Steiger J. H. (1980). Tests for comparing elements of a correlation matrix. Psychological Bulletin.

[B24] Watson D., Clark L. A., R. Hogan, J. Johnson, S. Briggs (1997). Extraversion and its positive emotional core. Handbook of personality psychology.

[B25] Webb T. L., Gallo I. S., Miles E., Gollwitzer P. M., Sheeran P. (2012). Effective regulation of affect: An action control perspective on emotion regulation. European Review of Social Psychology.

[B26] Webb T. L., Miles E., Sheeran P. (2012). Dealing with feeling: A meta-analysis of the effectiveness of strategies derived from the process of emotion model regulation. Psychological Bulletin.

[B27] Whiteside S. P., Lynam D. R. (2001). The five factor model and impulsivity: Using a structural model of personality to understand impulsivity. Personality and Individual Differences.

[B28] Williams A. D., Grisham J. R. (2012). Impulsivity, emotion regulation, and mindful attentional focus in compulsive buying. Cognitive Therapy & Research.

